# John Charnley Award: Preoperative Patient-reported Outcome Measures Predict Clinically Meaningful Improvement in Function After THA

**DOI:** 10.1007/s11999-015-4350-6

**Published:** 2015-07-23

**Authors:** Jonathan L. Berliner, Dane J. Brodke, Vanessa Chan, Nelson F. SooHoo, Kevin J. Bozic

**Affiliations:** Department of Orthopaedic Surgery, University of California, San Francisco, 500 Parnassus Avenue, MU 320-W, San Francisco, CA 94143 USA; Philip R. Lee Institute for Health Policy Studies, University of California, San Francisco, San Francisco, CA USA; Department of Orthopaedic Surgery, University of California, Los Angeles, Los Angeles, CA USA; Department of Surgery and Perioperative Care, Dell Medical School at the University of Texas at Austin, 1400 Barbara Jordan Blvd, Suite 1.114, Austin, TX 78723 USA

## Abstract

**Background:**

Despite the overall effectiveness of total hip arthroplasty (THA), a subset of patients remain dissatisfied with their results because of persistent pain or functional limitations. It is therefore important to develop predictive tools capable of identifying patients at risk for poor outcomes before surgery.

**Questions/purposes:**

The purpose of this study was to use preoperative patient-reported outcome measure (PROM) scores to predict which patients undergoing THA are most likely to experience a clinically meaningful change in functional outcome 1 year after surgery.

**Methods:**

A retrospective cohort study design was used to evaluate preoperative and 1-year postoperative SF-12 version 2 (SF12v2) and Hip Disability and Osteoarthritis Outcome Score (HOOS) scores from 537 selected patients who underwent primary unilateral THA. Minimum clinically important differences (MCIDs) were calculated using a distribution-based method. A receiver operating characteristic analysis was used to calculate threshold values, defined as the levels at which substantial changes occurred, and their predictive ability. MCID values for HOOS and SF12v2 physical component summary (PCS) scores were calculated to be 9.1 and 4.6, respectively. We analyzed the effect of SF12v2 mental component summary (MCS) scores, which measure mental and emotional health, on SF12v2 PCS and HOOS threshold values.

**Results:**

Threshold values for preoperative HOOS and PCS scores were a maximum of 51.0 (area under the curve [AUC], 0.74; p < 0.001) and 32.5 (AUC, 0.62; p < 0.001), respectively. As preoperative mental and emotional health improved, which was reflected by a higher MCS score, HOOS and PCS threshold values also increased. When preoperative mental and emotional health were taken into account, both HOOS and PCS threshold values’ predictive ability improved (AUCs increased to 0.77 and 0.69, respectively).

**Conclusions:**

We identified PROM threshold values that predict clinically meaningful improvements in functional outcome after THA. Patients with a higher level of preoperative function, as suggested by HOOS or PCS scores above the defined threshold values, are less likely to obtain meaningful improvement after THA. Lower preoperative mental and emotional health decreases the likelihood of achieving a clinically meaningful improvement in function after THA. The results of this study may be used to facilitate discussion between physicians and patients regarding the expected benefit after THA and to support the development of patient-based informed decision-making tools. For example, despite significant disease, patients with high preoperative function, as measured by PROM scores, may choose to delay surgery given the low likelihood of experiencing a meaningful improvement postoperatively. Similarly, patients with notably low MCS scores might best be counseled to address mental health issues before embarking on surgery.

**Level of Evidence:**

Level III, prognostic study.

## Introduction

THA generally reduces pain and improves function in patients with debilitating osteoarthritis of the hip. Despite the overall effectiveness of THA, a subset of patients experience persistent pain, functional limitations, and incomplete restoration of quality of life [1, 4, 38]. The proportion of patients who are dissatisfied with their outcomes after THA range from 7% to 15% [1, 25]. Furthermore, regional, racial, and sex variations in patient selection exist throughout the United States. These issues highlight the need for better defined surgical appropriateness and improved shared decision-making tools [12].

Recently, the focus of outcomes assessment has shifted away from physician-derived parameters to a more patient-centered analysis with the use of patient-reported outcome measures (PROMs) to evaluate pain, function, and quality of life. PROMs can be disease-specific or generic; each provides complementary information about a patient’s health-related quality of life and both can be used to assess the results of joint arthroplasty. Disease-specific measures such as the Hip Disability and Osteoarthritis Outcome Score (HOOS) are more sensitive to change within the context of a specific illness, whereas generic measures such as the SF-36 and SF-12 version 2 (SF12v2) capture a patient’s overall health including the effects of psychosocial health and medical comorbidities [19].

Preoperative pain and functional status among patients undergoing THA can predict pain and functional ability postoperatively [3, 11, 13, 22, 27, 30]. Patients who undergo THA with more baseline pain and poorer physical function experience a benefit of greater magnitude but with a lower absolute functional outcome than patients with less preoperative pain and disability [11, 13, 22]. Multiple studies have also demonstrated that poor mental and emotional health, as measured by generic PROMs, is correlated with poor functional outcomes, less pain relief, and patient dissatisfaction after THA [1–3, 11, 16, 36, 38]. Although disease-specific measures such as WOMAC and the related HOOS do not explicitly measure emotional health, evidence suggests that physical outcome measure scores are influenced by a patient’s psychological status [13, 16]. These findings suggest the importance of incorporating both the physical and mental components of preoperative PROMs into a decision-making tool. However, to our knowledge, this has not been done for patients undergoing THA.

The purpose of this study is to use prospectively collected SF12v2 and HOOS scores to define preoperative thresholds that predict a high probability of achieving meaningful clinical improvement 1 year after THA as defined by the minimal clinically important difference (MCID): (1) We hypothesize that threshold values will define maximum preoperative functional component scores that predict clinically meaningful improvements in functional outcomes; and (2) we further hypothesize that controlling for baseline mental and emotional health will modify and enhance the predictive ability of the threshold values.

## Patients and Methods

Data included in this study were obtained from a joint replacement outcomes registry maintained at the author’s institution. The registry includes patient-reported outcomes for patients undergoing THA collected preoperatively and at 1 year after surgery. The database also includes patient demographic information including age, sex, and race. Patients selected for this study had a history of primary unilateral THA with PROM data recorded at both preoperative and 1-year postoperative time points. To ensure the analysis was performed on a relatively homogenous patient population, the data analysis excluded patients with a diagnosis of pathological fracture, malignant neoplasm, or a history of a subsequent procedure on the operative hip.

All included patients had a history of osteoarthritis of the hip and underwent primary THA between 2009 and 2013. Five hundred thirty-seven patients met our inclusion criteria. This cohort represented 68% of the 793 patients undergoing primary, unilateral THA included in our institution’s joint replacement registry who had no subsequent revision procedure. The remainder were not included because they either did not have 1-year PROM scores available (38%) or were lost to followup completely (62%). The mean age of the patient cohort was 62 years (SD ± 13), 60% were female, and 80% were white.

SF12v2 and HOOS PROMs were collected preoperatively and 1 year postoperatively through either an electronic interface or on paper by a research assistant (DP). The SF12v2 is a modified version of the original SF-12 that uses the same 12 questions but with wording modifications to improve readability and ease of use. SF12v2 physical and mental composite scores (PCS and MCS, respectively) range from 0 to 100 in which a score of 0 indicates the lowest level of health and 100 indicates the highest level of health. The scores of both subscales are calculated from the survey’s 12 questions. The HOOS consists of 40 items and is scored from 0 to 100 with 0 being the worst level of pain and function. Preoperative and postoperative scores and SDs were determined using the scoring algorithms for each outcomes instrument. For the SF12v2 instrument, the PCS and MCS scales were used as separate outcomes. We anticipated that 500 patients would be included in the study, allowing the assessment of receiver operating characteristic (ROC) curves and areas under the curve (AUCs) with a precision of 0.03 for an expected AUC of 0.7.

The MCID is one way to define what constitutes a successful outcome after a surgical intervention. The concept of MCID has been defined as the smallest difference that patients perceive as beneficial [8]. MCID may be calculated using consensus, anchor, or distribution-based methods [24, 28]. The MCID after THA has been defined using the WOMAC and SF-36 instruments and can also be reliably estimated as half the SD of outcome scores for a given instrument [7, 31, 34, 42]. MCID values were calculated separately for the SF12v2 PCS, SF12v2 MCS, and HOOS as half the SD of mean change scores for that specific PROM. We used the distribution-based method to estimate MCID given its relative convenience when compared with the generally preferred anchor and consensus-based methods [31]. Anchor-based methods require a separate subjective assessment measure of a patient’s perceived benefit from an intervention, data that were not collected by our institution’s joint replacement registry [43]. The calculated MCID value was 4.6 for the SF12v2 PCS, 6.0 for the SF12v2 MCS, and 9.1 for the HOOS. Overall, 77% of patients achieved improvement greater than the MCID on the SF12v2 PCS, 41% on the SF12v2 MCS, and 93% on the HOOS after unilateral primary THA.

Optimal threshold values for each outcomes instrument (SF12v2 PCS, SF12v2 MCS, and HOOS) were determined by a nonparametric ROC analysis. The Youden index, which maximizes the balance of sensitivity and specificity, was used to calculate threshold values [44]. The c-statistic (AUC) of this ROC analysis indicated the predictive validity of this binary classifier test for predicting a patient would achieve the MCID. Predictive models are considered reasonable if the AUC is greater than 0.7 and excellent if greater than 0.8 [17]. For this study, AUC values greater than 0.7 were considered acceptably predictive.

A two-stage hierarchical multivariable logistic regression analysis was performed. First, logistic regression was performed to determine the relative influence of preoperative MCS score on patients’ likelihood of achieving the MCID based on their preoperative PCS or HOOS scores. This analysis was necessary to control and adjust for individual patient preoperative variability to allow for comparisons between patients’ clinically meaningful improvements. Subsequently, new Youden thresholds for PCS and HOOS were calculated from the fitted logistic regression equation of the predicted probability of obtaining the MCID, generating a new threshold value for each potential preoperative MCS score. These new threshold values were then used to calculate new c-statistics to determine changes in the predictive ability of the PCS and HOOS threshold values after controlling for preoperative MCS scores.

## Results

**Fig. 1A–B:**
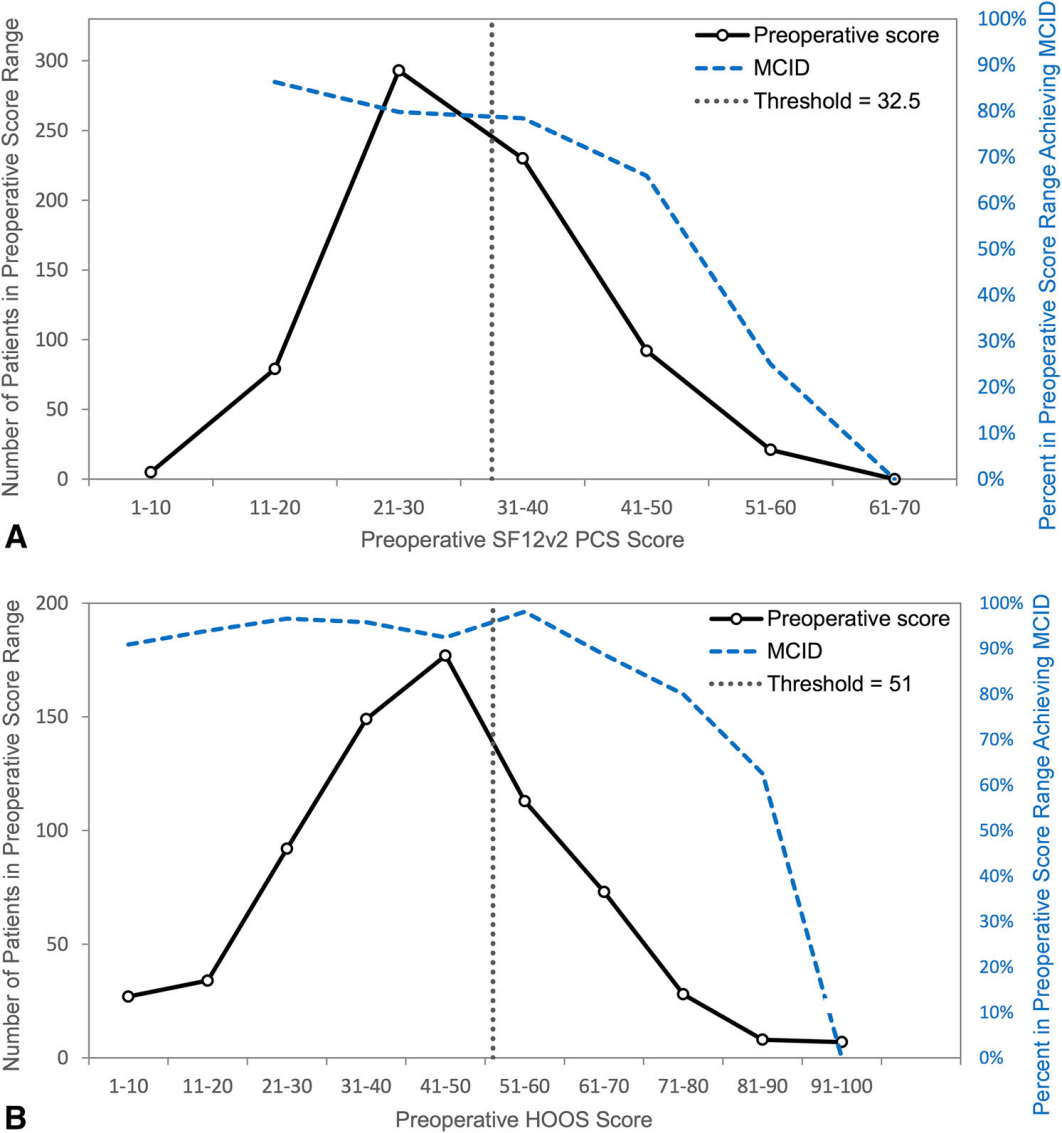
The calculated threshold values, indicated by the dotted vertical lines, do not represent true cutoffs but instead serve to represent points after which a patient’s likelihood of experiencing a clinically meaningful improvement in function begins to more rapidly diminish. (**A**) The SF12v2 PCS threshold value of 32.5 was not acceptably predictive of a patient’s likelihood of experiencing a clinically meaningful improvement in outcome as measured by the 1-year postoperative SF12v2 PCS score (AUC 0.62). (**B**) The HOOS threshold value of 51 was acceptably predictive of a patient’s likelihood of experiencing a clinically meaningful improvement in outcome as measured by the 1-year postoperative HOOS score (AUC 0.74).

The calculated threshold values for functional outcome measures SF12v2 PCS and HOOS defined a maximum preoperative score after which a patient’s likelihood of experiencing a clinically meaningful improvement in functional outcome from THA, as defined by the MCID, began to diminish. The threshold values for SF12v2 PCS and HOOS were 32.5 and 51.0, suggesting that patients with preoperative PROM scores above these values were less likely to experience a minimum clinically important difference. A threshold value was also generated for the SF12v2 MCS score. The corresponding sensitivity and specificity values for each threshold ranged from 54% to 76% (Table 1). The SF12v2 PCS threshold was not acceptably predictive with an AUC value of 0.62 (Fig. 1A). The HOOS threshold value of 51.0 proved to be acceptably predictive of a patient’s likelihood of achieving the MCID with an AUC value of 0.74 (Fig. 1B).

**Table 1:** Threshold values for univariate and multivariate analysis

Score	Threshold(s)	AUC	Sensitivity	Specificity	p value
SF12v2 PCS	< 32.5	0.62	65%	54%	< 0.001
SF12v2 MCS	< 48.0	0.83	76%	74%	< 0.001
HOOS	< 51.0	0.74	70%	64%	< 0.001
Multivariate SF12v2 MCS/SF12v2 PCS*	See Fig. 2A	0.69	68%	66%	< 0.001
Multivariate SF12v2 MCS/HOOS*	See Fig. 2B	0.77	80%	61%	0.003

* Also controlled for gender, age, and race; AUC = area under the curve; SF12v2 = SF-12 version 2; PCS = physical component summary; MCS = mental component summary; HOOS = Hip Disability and Osteoarthritis Outcome Score.

**Fig. 2A–B:**
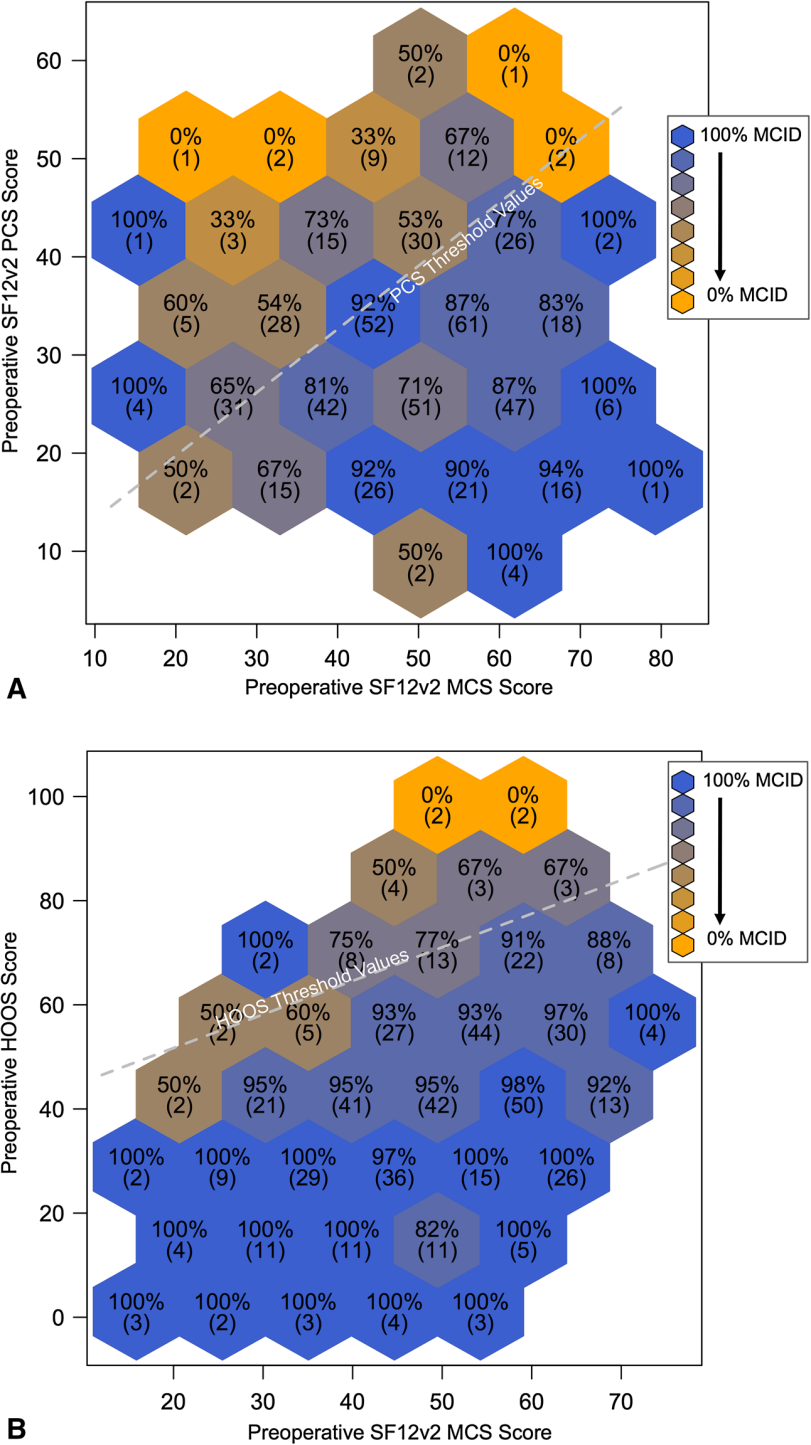
SF12v2 PCS and HOOS threshold values (represented by dashed lines) are dependent on preoperative MCS score and demonstrate a linear relationship. Postoperative data are plotted in a binned fashion, which demonstrates the likelihood of attaining a MCID across different preoperative PROM score combinations. Hexagonal cells are labeled and shaded according to the proportion of patients within that cell who obtained the MCID (absolute number of patients in parentheses). By situating patients within a specific bin, one is able to visualize an approximate likelihood of obtaining a MCID based on preoperative PROM scores in the context of calculated threshold values. (**A**) After adjusting for preoperative mental and emotional health, SF12v2 PCS threshold values demonstrated an improved predictive ability (AUC 0.69), yet remained below the acceptably predictive value of 0.70. (**B**) The predictive ability of HOOS threshold values improved from 0.74 to 0.77 after adjusting for preoperative mental and emotional health.

The predictive ability of both the HOOS and SF12v2 PCS threshold values improved after adjusting for preoperative mental and emotional health with a multivariate analysis. The HOOS c-statistic improved from 0.74 to 0.77 and the SF12v2 PCS c-statistic improved from 0.62 to 0.69 (Table 1). For each potential preoperative MCS score, ranging from 0 to 100, a new SF12v2 PCS and HOOS threshold value was calculated from the fitted logistic regression equation. Higher preoperative SF12v2 MCS scores resulted in higher threshold values for both SF12v2 PCS (Fig. 2A) and HOOS (Fig. 2B) with each 10-point increase in preoperative SF12v2 MCS score resulting in an approximate 6-point increase in both HOOS and SF12v2 PCS threshold values. This suggests that patients with better mental and emotional health are more likely to experience a clinically meaningful improvement in functional outcome despite having higher baseline function. Taken together, these results indicate that patients with better baseline mental and emotional health (higher preoperative MCS scores) and worse preoperative function have the highest probability of experiencing a clinically meaningful improvement in function after THA.

## Discussion

For both the patient and physician, the decision to proceed with THA is complex and multifactorial. Although the majority of patients experience meaningful clinical improvement after THA, a subset of patients do not [1, 6, 9, 23, 27, 30]. To improve patient satisfaction and outcomes, both appropriateness criteria and shared decision-making tools should be improved. Although prior studies have attempted to define explicit clinical criteria for the appropriateness of THA, the subjective nature of the procedure’s indications requires patients to weigh the risks and benefits on the basis of their own values [10, 33]. To the authors’ knowledge, this study is the first to use baseline functional status, adjusted for mental and emotional health, to predict which patients are most likely to experience a clinically meaningful improvement in function after THA. The results of this study define a preoperative HOOS threshold value that is capable, with acceptable predictive ability, of differentiating patients more likely to experience the MCID from those who are less likely. Furthermore, this HOOS threshold value has been shown to vary and become more predictive when taking into account a patient’s preoperative mental and emotional health.

This study has several limitations. This study was performed at a single institution and accordingly, the results may not be applicable to patients who are underrepresented in our study population. Because of cultural and societal differences, our results in a predominantly white, North American population may not reflect those elsewhere. However, both HOOS and SF12v2 have demonstrated good applicability across populations, and although specific threshold values may differ, we believe that our findings can be generalized. Additionally, we believe that the methods described in this study can be applied to surgeon-specific data with the application of a computational algorithm to generate threshold values that can be used to inform patient-specific shared medical decision-making. Such an algorithm may be incorporated into joint replacement registry applications and therefore have broad implications with limited barriers to entry.

The 1-year followup may be regarded as a limitation. However, we feel that 1-year followup was appropriate given the objective of our study and supporting evidence from previous literature related to time to full recovery after total joint arthroplasty as measured by patient-reported outcomes. Specifically, the greatest change with regard to pain, function, and mental health has been shown to occur within the first 6 months after surgery [13, 35, 37]. Two hundred fifty-six (38%) of the patients from our institution’s joint replacement registry who met the original inclusion criteria during the years 2009 to 2013 were not included in the study. This included 158 who were lost to followup and 98 who did have postoperative followup but not at the 1-year time point. Importantly, no differences were found between the study cohort and all patients lost to followup when comparing preoperative PROM scores (Table 2). Similarly, no significant difference in postoperative HOOS scores was found between the study cohort and patients not included in the study as a result of missing 1-year data.

**Table 2:** Comparison of study cohort to patients without 1-year PROM data

Comparison	Study cohort	Missing 1-year data	p value*
Number of patients	537	256	
Preoperative HOOS^†^	43.4 (17.5)	41.0 (18.5)	0.155
Preoperative SF12v2 PCS	30.8 (9.5)	28.7 (8.4)	0.453
Preoperative SF12v2 MCS	48.5 (12.3)	47.1 (13.5)	0.802
Postoperative HOOS	83.1 (18.2)	78.0 (21.5)^‡^	0.146
Postoperative SF12v2 PCS	43.6 (12.2)	39.8 (12.3)^‡^	0.001
Postoperative SF12v2 MCS	51.7 (10.5)	51.2 (10.6)^‡^	0.175

* To compare the mean PROM scores of the study cohort to the mean PROM scores of those without 1-year data, a Student’s t-test was used.

^†^mean (SD).

^‡^includes patients with at least one postoperative PROM score available (but missing 1-year PROM data); this group represented 38% (n = 98) of the total patients not included in the study cohort; postoperative PROM scores included in the mean were those available from latest followup; this ranged from 2 to 4 years postoperatively; PROM = patient-reported outcome measure; HOOS = Hip Disability and Osteoarthritis Outcome Score; SF12v2 = SF-12 version 2; PCS = physical component summary; MCS = mental component summary.

Previously published MCID values for the SF-36 and WOMAC instruments in the setting of THA are variable and an ideal means of calculating MCID with regard to a specific intervention remains to be determined [8, 15, 28, 34, 41]. The value of a MCID is ultimately defined by what is interpreted as important to a patient and is therefore not a fixed attribute. Differing patient populations, length of patient followup, and methods of calculation lead to variability in reported MCID values with each method having its own potential shortcomings. This study used a distribution-based method that, although widely used, is generally not a preferred method as a result of several limitations. Distribution-based methods are based purely on statistical reasoning and therefore do not include actual patient assessments of their condition. Instead, they are able to determine an effect or outcome that is unlikely to be attributable to random measurement error. Statistically significant changes or noteworthy effect sizes at the group level may not be significant at the individual level [21]. In the current study, every attempt was made to control for individual variability using multivariate techniques. Prior studies designed to estimate the MCID for WOMAC after THA using anchor-based methods have found consistently higher values than those using distribution-based methods [7, 34, 40]. This may suggest that distribution-based methods underestimate the amount of postoperative improvement necessary to be meaningful for patients.

Given the limitations of our institution’s joint replacement registry, similar to other regional and national joint replacement registries in the United States, we did not have access to a subjective patient assessment of improvement and were therefore unable to perform an anchor-based method. However, given the fact that MCID values can be sample-specific, we favored a method that used data from our study population over adopting MCID values defined in previous studies. Applicable to any study that uses MCID, the reader should be made aware of the associated limitations and the resulting impact on its clinical applications.

The definition of a “successful” outcome is a controversial issue. For the purpose of our study it is defined as attaining a MCID in the context of specific PROMs, which may not be the best definition of success. This definition excludes patient satisfaction, which is a separate outcome and has been shown to be highly dependent on preoperative patient expectations [32]. The selection of threshold values using Youden’s index, which maximizes the combined sensitivity and specificity of the cutoff point, may not be the most clinically relevant method. This may explain the relatively high proportion of patients in our study that fall outside of the defined thresholds when compared with prior studies of THA use and appropriateness based on clinical criteria [10, 33]. When considering the likelihood of meaningful clinical improvement after THA, clinicians may prefer thresholds with higher sensitivity at the expense of specificity, thus detecting more patients with problems at the expense of falsely identifying healthy patients as troubled. Thus, our threshold values should not be regarded as appropriate use criteria, but rather as predictive tools for patient education and shared decision-making.

Using the HOOS, a disease-specific PROM, we identified a preoperative threshold value that predicts clinically meaningful improvement in functional outcome after primary THA. The threshold value for HOOS, which was a maximum of 58 out of a possible 100 points, was sufficiently predictive of attaining a MCID (AUC, 0.74). This suggests that patients with higher baseline HOOS scores are progressively less likely to experience a clinically meaningful improvement after surgery, a trend of diminishing returns that has been previously described [22]. Our findings are consistent with prior evidence suggesting that preoperative pain and functional status are predictive of functional ability after THA [3, 5, 11, 13, 22, 27, 30]. However, when we used the SF12v2 PCS, a generic PROM, we found that the threshold value was not acceptably predictive. When compared with disease-specific measures, generic PROMs such as the SF12v2 have been shown to be less sensitive to changes in health after THA [29]. This likely explains the difference between HOOS and SF12v2 PCS threshold values’ predictive abilities. To our knowledge, no prior study has attempted to determine preoperative threshold values that are sufficiently predictive of functional improvements after primary THA. A prior study assessed the ability of preoperative Oxford Hip Scores to predict patient satisfaction at 6 months postoperatively [26] but did not address the effect of preoperative mental and emotional health on patient satisfaction. The authors found no correlation between preoperative Oxford scores and patient satisfaction. Preoperative thresholds were also calculated using ROC analysis and demonstrated poor predictive ability. When considered in the context of the current study, these results suggest that although preoperative PROM scores may be sufficiently predictive of postoperative function, this does not correlate with patient satisfaction. This finding is consistent with prior literature, which demonstrates that preoperative pain and function are not associated with satisfaction after surgery [14, 20].

A preoperative SF12v2 MCS threshold value was also calculated and assessed for its ability to predict improvements in postoperative MCS scores. Although this threshold value exhibited the largest c-statistic in our univariate analysis (AUC, 0.83), only 41% of patients in this study obtained the MCID for SF12v2 MCS. The ability of THA to improve a patient’s mental and emotional health has been questioned with many suggesting that it is not an effective intervention in this regard [35]. For this reason, we believe that the maximum threshold value for SF12v2 MCS is not clinically relevant and we do not suggest that a patient be discouraged from having surgery as a result of concern that they will not experience a sufficient mental health improvement after THA.

The predictive ability of both SF12v2 PCS and HOOS threshold values improved after controlling for baseline mental and emotional health, as quantified by preoperative SF12v2 MCS scores. Additionally, baseline SF12v2 MCS scores paralleled functional threshold values. These findings are consistent with prior evidence, which demonstrates that poorer baseline mental and emotional health is associated with smaller improvements in function after THA [3, 27, 36, 38]. By comparison, patient comorbidities and age have little effect on PROM scores after THA [11, 18].

For both physicians and patients, proceeding with THA is a complex decision influenced by social, functional, and psychological factors. These have been difficult to quantify until the recent adoption of PROMs, which focus more on patients’ experience and less on physician direction. Shared decision-making tools such as PROMs have been shown to improve patient-provider communication, help patients reach decisions that are better aligned with their personal values, and result in higher satisfaction after total joint arthroplasty [39]. PROMs may also allow patients to take their own score information and get a sense of what to expect after surgery and engage with their providers in the decision-making process. Furthermore, physicians may use these data to identify the subset of patients with preoperative PROM scores that place them at a low likelihood of experiencing a clinically meaningful benefit such as patients with high preoperative function or those with poor mental and emotional health, which could facilitate further discussions surrounding the timing of surgery or the need for additional preoperative interventions. Importantly, we do not suggest that our threshold values be considered true appropriate use criteria. Rather, they should be considered as a general framework to interpret preoperative PROM scores and implement them as a predictive tool.

We anticipate that similar methodology will be applied using national joint replacement registry data to develop patient-specific decision aids for THA. Future studies are needed to assess the ability of preoperative PROMs to affect the clinical decision-making process for patients with advanced hip osteoarthritis and to improve postoperative patient satisfaction and outcomes.
